# Patients with Nipple-Areola Paget’s Disease and Underlying Invasive Breast Carcinoma Have Very Poor Survival: A Matched Cohort Study

**DOI:** 10.1371/journal.pone.0061455

**Published:** 2013-04-19

**Authors:** Hong Ling, Xin Hu, Xiao-Li Xu, Zhe-Bin Liu, Zhi-Min Shao

**Affiliations:** 1 Department of Oncology, Shanghai Medical College, Fudan University, Shanghai, China; 2 Department of Breast Surgery, Fudan University Shanghai Cancer Center/Cancer Institute, Shanghai, China; 3 Department of Pathology, Fudan University Shanghai Cancer Center/Cancer Institute, Shanghai, China; University of North Carolina School of Medicine, United States of America

## Abstract

Paget’s disease (PD) of the breast is a rare disease. The survival rate of PD was reported to depend on the characteristics of the underlying carcinoma. This study aimed to investigate the characteristics and survival rate of PD patients with underlying invasive breast carcinoma (IBC). Fifty-two patients were diagnosed with PD and an associated IBC from 2001 to 2005 in Fudan University Shanghai Cancer Center. Twenty-four (46.2%) had no clinical manifestation of PD and were diagnosed unexpectedly by a histologic examination. The 52 patients were all recruited in this study as the PD group. They tended to have greater chances of lymph node involvement (53.8% vs. 35.7%), lower hormone receptor expression (34.6% vs. 69.7%), higher human epidermal growth factor receptor 2 (HER2) expression (76.9% vs. 21.3%), and worse survival (5-year relapse-free survival (RFS) 52.2% vs. 86.7%, P<0.01; breast cancer-specific overall survival (OS) 62.1% vs. 91.8%, P<0.01) when compared with patients diagnosed with IBC. A matched study was then performed to investigate whether the poor survival of patients in the PD group was due to the unfavorable prognosis of the underlying IBC. One hundred and fifty-six (3∶1 ratio of controls to PD patients) patients diagnosed with IBC only were recruited into the matched group. The match was conducted according to four variables: dimension of IBC, lymph node status, hormone receptor status and HER2 status. The 5-year RFS (52.2% vs. 81.4%, P<0.01) and OS (62.1% vs. 85.9%, P<0.01) were both lower for patients in the PD group than those in the matched group. Patients with PD and underlying IBC had poor survival. Their survival was worse than that of patients with IBC of similar stage and characteristics. For patients with no clinical PD manifestation who were histologically diagnosed as PD, survival might be worse compared to patients with clinically diagnosed PD.

## Introduction

Paget’s disease (PD) is a very rare breast disease. The incidence has been reported to be 0.5–5% of all diagnosed breast cancers [1]–[6]. PD is pathologically characterized by the infiltration of the nipple epidermis by large, clear, ballooned cells, now recognized as malignant breast epithelial cells, which cause an eczematoid eruption on the nipple and areola. PD of the breast typically clinically presents as a skin alteration in the nipple-areola area. This nipple-areolar skin change was first reported by Velpeau in 1856 [7]. In 1874, Sir James Paget described this change as “an eczematous change in the skin of the nipple preceding an underlying mammary cancer” [8]. The prevalence of an associated cancer ranges from 67–100%, with most studies reporting over 90% [9]–[10].

Three prognostic factors for PD have been reported in different studies to date: (1) a palpable mass on presentation; (2) an underlying invasive carcinoma of the breast; and (3) the status of the axillary lymph nodes (ALN) [9]–[12]. Almost 90% of patients who had a palpable mass will have an underlying invasive carcinoma. Conversely, 66–86% of patients without a clinical mass will have ductal carcinoma in situ (DCIS) alone. The survival rate for PD with carcinoma in situ is better than that for PD with invasive carcinoma. Therefore, the authors believe the prognosis of PD is mostly determined by the pathologic stage of the associated carcinoma although this hypothesis has not been proved by case-control studies. Due to the limited number of patients with PD, case-control studies have been very rare. In the daily clinical practice of Fudan University Shanghai Cancer Center (FUSCC), physicians found that patients diagnosed as PD with underlying invasive carcinoma might have much worse survival than patients diagnosed with invasive carcinoma of the same stage. This finding was a challenge to the traditional knowledge of PD. The purpose of this study was to demonstrate this finding by investigating the prognosis of patients with PD and an underlying invasive carcinoma via a cohort-matched study.

## Materials and Methods

### Patient Selection

The patients were chosen from the database of FUSCC, China. The database included all of the patients who underwent operations between 2001 and 2005 in FUSCC. Women were only eligible for enrollment in this study if all of the following criteria applied: (1) received mastectomy (with nipple-areola removed); (2) had histologically confirmed invasive carcinoma; (3) had adequate ALN evaluation and treatment (ALN dissection or sentinel lymph node biopsy followed by dissection if positive). Women were excluded if any of the following criteria applied: (1) neo-adjuvant therapy received prior to surgery and/or (2) a prior history of other malignancy. Our study was approved by the independent ethical committee/institutional review board of FUSCC (Shanghai Cancer Center Ethical Committee). All patients have signed written informed consent.

All patients diagnosed with nipple-areola PD with underlying IBC were recruited as part of the study group (PD group). Because their underlying carcinomas were all ductal carcinomas, only patients diagnosed with invasive ductal carcinoma (IDC) without PD were recruited into the control group for further comparison. Seven hundred patients (140 consecutive patients per year) were recruited for the control group.

Because the pathologic comparison (see results) showed that tumors in the PD group expressed more unfavorable prognostic factors than those in the control group, a matched study was performed to compare the survival of patients in the PD group with that of patients with IDC of similar prognostic factors. The match was conducted according to four variables: dimension of IDC, lymph node status, hormone receptor (HR) status and human epidermal growth factor receptor 2 (HER2) status. The matched group was also derived from the database. After matching, controls were randomly selected in a strict 3∶1 ratio to patients in the PD group.

### Pathology Data

As part of the routine clinical practice of FUSCC, the breast removed due to mastectomy was carefully reviewed, including tumor, normal breast tissue of other quadrants, nipple-areola area, etc. We collected the complete pathology data including pathology type, tumor diameter, grade, and estrogen receptor (ER), progesterone receptor (PR), and HER2 status. Tumors that were either ER or PR positive were considered HR positive.

### Follow-up

The patients treated at the FUSCC were all instructed to attend follow-up visits after the operation. Follow-up via phone was performed if the patient did not attend her appointment. The time and site of the first detected relapse were recorded, as well as the time and cause of death.

### Statistical Methods

The match was performed according to the four variables mentioned above with SAS 8.2 (SAS, NC, USA). The main endpoints were the first relapse (relapse-free survival, RFS) and mortality due to breast cancer (breast cancer-specific overall survival, OS). The significance of any correlation was assessed by the chi-squared test. Survival curves were constructed using the Kaplan-Meier technique. All of the statistical analyses and curves were completed using SPSS 17 (SPSS, IL, USA).

## Results

### General Information for the PD Group

A total of 52 patients were diagnosed with nipple-areola PD with underlying IBC during the study period and recruited into the PD group. The median age of these patients was 50 years, with a range from 27 to 86 years. Twenty-two (42.3%) patients’ primary symptoms were skin change in the nipple-areola area, 5 (9.6%) patients’ primary symptom was nipple discharge, and the remaining 25 (48.1%) patients came to see the doctor because of a mass in the breast or axilla.

Postoperative adjuvant treatment was designed according to the current National Comprehensive Cancer Network (NCCN) guidelines and the judgment of the physicians. Hormone therapy was administered to patients positive for hormone receptor after adjuvant chemotherapy/radiotherapy. Targeted therapy was very rarely used during that period because of its high cost in China at that time. The main clinical and pathological characteristics and the adjuvant treatment of patients in this study are shown in Table 1.

**Table 1:** Patients’ Characteristics and the Adjuvant Treatment Received.

	PD group (n = 52)	Control Group (n = 700)		Matched Group (n = 156)	
			Significanceξ		Significanceξ
Age			P = 0.87		P = 0.42
≥50	27 (51.9%)	372 (53.1%)		71 (45.5%)	
<50	25 (48.1%)	328 (46.9%)		85 (54.5%)	
Dimension			P<0.01		/
≤2 cm	17 (32.7%)	423 (60.4%)		51 (32.7%)	
>2 cm	35 (67.3%)	275 (39.3%)		105 (67.3%)	
unknown		2 (0.3%)			
Grade			P = 0.01		P = 0.63
I	4 (7.7%)	120 (17.1%)		17 (10.9%)	
II	21 (40.4%)	355 (50.7%)		53 (34.0%)	
III	27 (51.9%)	225 (32.1%)		86 (55.1%)	
ALN			P<0.01		/
positive	28 (53.8%)	250 (35.7%)		84 (53.8%)	
negative	24 (46.2%)	450 (64.3%)		72 (46.2%)	
Hormone Receptor			P<0.01		/
positive	18 (34.6%)	488 (69.7%)		54 (34.6%)	
negative	34 (65.4%)	212 (30.3%)		102 (65.4%)	
Her2			P<0.01		/
positive	40 (76.9%)	149 (21.3%)		120 (76.9%)	
negative	12 (23.1%)	551 (78.7%)		36 (23.1%)	
Chemotherapy			P = 0.02		P = 0.85
None	7 (13.5%)	151 (21.6%)		19 (12.2%)	
CMF	4 (7.7%)	93 (13.3%)		15 (9.6%)	
Anthracycline- based	29 (55.7%)	376 (53.7%)		78 (50.0%)	
Taxane-based	12 (23.1%)	68 (9.7%)		42 (26.9%)	
Unkown	0	12 (1.7%)		2 (1.3%)	
Radiation			P<0.01		P = 0.62
No	22 (42.3%)	445 (63.6%)		59 (37.8%)	
Yes	30 (57.7%)	238 (34%)		95 (60.9%)	
Unkown	0	17 (2.4%)		2 (1.3%)	
Endocrine Therapy			P<0.01		P = 0.93
No	34 (65.4%)	212 (30.3%)		103 (66.0%)	
Yes	18 (34.6%)	480 (68.6%)		53 (33.9%)	
Unkown	0	8 (1.1%)		0	
Target Therapy			P = 0.02		P = 0.82
No	50 (96.2%)	695 (99.3%)		151 (96.8%)	
Yes	2 (3.8%)	5 (0.7%)		5 (3.2%)	

ξ Compared with PD group.

The median length of follow-up was 59 months (range, 5∼129 months). During the follow-up period, 23 patients developed recurrence or metastasis, and 18 breast cancer-specific deaths occurred. For patients with nipple-areolar skin alteration as their primary symptom, the 5-year RFS was 63.6%; for patients with breast/axillary mass as the primary symptom, the 5-year RFS was 34.8% (p = 0.09).

### PD Group vs. Control Group

**Figure 1:**
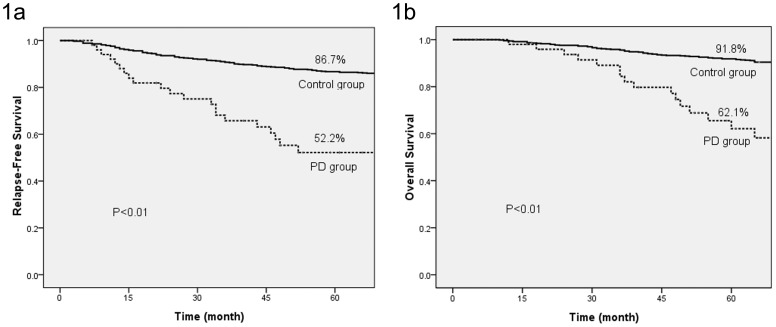
5-year relapse free survival (a) and breast cancer-specific overall survival (b) of PD group and control group.

Compared with the control group, tumors in the PD group had a larger tumor size, a greater chance of ALN involvement, lower HR expression and higher HER2 expression. The follow-up data showed that the patients in the PD group had a worse 5-year RFS (52.2% vs. 86.7%, P<0.01) and breast cancer-specific OS compared to patients in the control group (Fig. 1).

### PD Group vs. Matched Group

**Figure 2:**
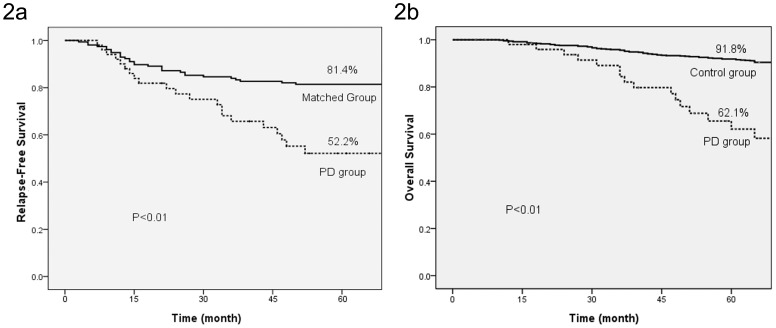
5-year relapse free survival (a) and breast cancer-specific overall survival (b) of PD group and matched group.

One hundred and fifty-six patients were recruited into the matched group. The 5-year relapse-free survival and breast cancer-specific overall survival were both lower for patients in the PD group compared to the matched group (Fig. 2).

### “Occult” Paget’s Disease

Patients noted on physical examination of the breast to have nipple or areolar eczema, ulceration, moist erythema, chronic vesicles, granular erosions or nipple discharge were categorized as having clinical manifestations of PD (“clinical PD”). Twenty-four (46.2%) patients had no clinical PD before operation; their PD was found unexpectedly by routine nipple-areola histologic examination after mastectomy. We call such cases as “occult” PD in this study. Patients with “occult” PD tended to have a worse 5-year RFS than patients with clinical PD do (38.0% vs. 63.0%, p = 0.09). The 5-year OS for patients with “occult” PD and clinical PD were 54.2% and 81.1%, respectively (p = 0.13).

## Discussion

Mastectomy is the only standard operation for patients with clinical PD at FUSCC because of the nipple involvement. Therefore, patients who received breast-conservative surgery were excluded from the control group and the matched group to avoid bias. Additionally, mastectomy was more acceptable to patients than breast-conservative surgery in China in the early 2000s due to the fear of recurrence and limited medical resources (e.g., lack of radiation facilities). At FUSCC, over 85% of patients diagnosed with IBC received a mastectomy at that time. Thus, some patients with “occult” PD were diagnosed unexpectedly by routine pathological examination of the nipple-areola area.

A total of 52 patients with PD and associated IBC were investigated: 73% of them had a palpable breast mass, and 54% of the 52 patients had ALN involvement. These findings are in agreement with most of the literature; the incidence of ALN involvement for patients with PD and a palpable mass was approximately 60%, and Kollmorgen and associates reported an incidence of ALN involvement of 57% [11]. Of the patients in our study, 76.9% had tumor overexpression of HER2. This proportion is comparable to other reported values. Caliska’s study showed that 83.3% of patients’ invasive tumors were HER2 positive [12]. Wolber demonstrated HER2 overexpression in 79% of cases of invasive carcinoma associated with PD [13]. In the Kothari study, 82.5% of such patients had HER2-positive tumors [14].

Forty-six percent of patients in this study had no clinical PD. Most studies have not pay a great deal of attention to this type of “occult” PD. Because there is no sign of nipple disorder before the operation, it is easy to miss if the pathologist does not examine sections of the nipple very carefully. In Kollmorgen’s study, 12 (15%) patients had no clinical PD. It should be noted that PD had been reported by several investigators as the first local recurrence event after breast-conservative surgery or nipple-sparing mastectomy. Peterse reported 2 cases of PD after breast-conservative surgery, which accounted for 13.3% of the observed local recurrence [15]. Menzies and Plastara also reported PD as the first recurrence after conservative surgery [16]–[17]. PD has also been found after nipple-sparing mastectomy. Lohsiriwat reported 7 cases of PD as a recurrence after nipple-sparing mastectomy, which accounted for 19.4% (7/36) of total recurrences [18]. Our institute had two similar cases. One patient developed PD as a first recurrence 1.5 years after nipple-sparing mastectomy, and the other developed PD 2 years after conservative surgery. Considering the short disease-free interval after the operation and the fact that almost no mammary gland was left after nipple-sparing mastectomy, we believe that the PD already existed before the operation. These cases might be the above-mentioned “occult” PD and might have been misdiagnosed before operation.

Patients with PD with an underlying breast mass have been shown to have poor survival. The 5- and 10-year survivals rates have been reported to be 32∼43% and 31∼49%, respectively. As explained above, a lesion with a palpable mass usually reflects invasive disease. The latest studies reported that the 10-year breast cancer-specific survival of PD with underlying invasive carcinoma was 75% [19]. The results of our study were in agreement with the literature values. The 5-year breast cancer-specific survival of the patients in our PD group was 62.1%.

Was the poor survival of patients in the PD group due to the relatively late clinical stage and the overexpression of unfavorable prognostic factors? This question can only be answered by a matched cohort study. Due to the limited number of patients with PD, case-control studies have been very rare. Kothari collected 40 patients with PD and underlying IBC and compared the survival of these 40 patients with 120 patients who had IBC only after matching for age, tumor size, grade, and nodal status. That study demonstrated that patients with PD and IBC had a significantly worse prognosis (10-year OS 49%) compared to those with IBC only (64%). These findings were in line with those of our study. Kothari then attributed the poor survival of patients with PD with associated IBC to the high HER2 positivity. To address this question, he further compared the survival of the 40 patients with PD with the survival of 40 IBC patients after matching for age, tumor size, grade, nodal status and HER2 status. Once HER2 status was controlled for, the study group had a similar overall survival rate to matched controls. Our study was also a case-control study. We selected four widely recognized prognostic factors for patient matching, including HER2 status. We demonstrated that patients with PD with IBC had worse survival than those with IBC only. This result was different from Kothari’s study and might be due to different patient inclusion criteria and different variables used for matching in the two studies. Kothari only included patients with clinical PD and excluded those with “occult” PD from his study, but we kept the latter patients. Patients with “occult” PD tended to have worse survival than those with clinical PD in our study. The difference in survival was of borderline statistical significance (p = 0.09), mostly due to the limited case number. Considering the inclusion of patients with “occult” PD, it is reasonable that the survival of the study group in our study was worse than in Kothari’s. Additionally, the variables for matching were different between Kothari’s study and ours. We used hormone receptor status as a variable, and that study used grade and age as variables. However, there was no significant difference in grade or age between the two groups in our study.

The pathogenesis of PD remains debatable. There are two theories about the origin of Paget’s cells. The transformation theory proposes that Paget’s cells are transformed in situ keratinocytes of the epidermis of the nipple. The migration theory is more widely accepted; it assumes that Paget’s cells are ductal carcinoma cells that have migrated from the underlying mammary ducts to the epidermis of the nipple. It seems that both theories could be supported by our study. Patients who had nipple disorder as a primary symptom developed a mass in their breast several months or years later. This occurrence can be explained by the transformation theory. Under this explanation, the epidermis of the nipple-areola developed PD first and the carcinoma then invaded into the ducts and formed a breast mass. For the patients who had a breast mass identified first, the nipple disorder might appear later or they may not develop clinical PD. The migration theory works well in this type of patient. The ductal carcinoma appears first, and the carcinoma cells then migrate through the duct into the nipple and become PD. Some of these patients received surgery before the clinical PD symptoms appeared, and we defined these patients as having “occult” PD. Because the pathogenesis of these two types of PD is different, their prognoses might also be different. However, this is only our hypothesis, and further investigations are required to evaluate it. Considering the rare incidence of PD, it is difficult to prove any hypothesis about the disease.

### Conclusions

Patients with PD and underlying IBC tended to have a greater chance of lymph node involvement, lower hormone receptor expression, higher HER2 expression and worse survival compared to those without PD. The subsequent matched study confirmed that the survival of patients with PD and underlying IBC was reduced compared to patients with IBC with similar prognostic factors (stage and characteristics). This finding suggests that PD itself is an indicator of poor survival. Some patients were pathologically diagnosed as PD in the absence of clinical PD manifestation. They might have a worse prognosis than patients with clinical PD. Pathologists should carefully examine the nipple after mastectomy to diagnose such non-clinical “occult” PD. Additionally, further studies need to be performed to investigate the characteristics of “occult” PD.
